# Carbon emission reduction and hydrogen production maximization from carbon emission-based hydrogen sources

**DOI:** 10.1007/s11356-024-34229-2

**Published:** 2024-07-12

**Authors:** Johnson Kehinde Abifarin, Fredah Batale Abifarin

**Affiliations:** 1 School of Engineering, College of Engineering, Computing, and Cybernetics, Australian National University, Canberra, Australia; 2 Department of Metallurgical and Materials Engineering, Ahmadu Bello University, Zaria, Nigeria

**Keywords:** Energy vector, Clean production, Carbon capturing, Taguchi grey relational analysis, Optimization, Sustainable energy

## Abstract

This study aims to optimize hydrogen (H_2_) production via ethanol steam reforming (ESR) and water gas shift reaction (WGSR) pathways, focusing on minimizing CO, CO_2_, and CH_4_ emissions while maximizing H_2_ yield. Employing Taguchi grey relational analysis, we investigate the intricate balance between production conditions and multi-response gas generation. Utilizing Origin Pro software, regression modeling forecasts individual and overall gas generation. Our analysis identifies optimal conditions: a feed liquid flow rate of 2 mL/min, water-to-carbon ratio of 3, ESR temperature of 300 °C, and WGSR temperature of 350 °C. These conditions promise clean, efficient H_2_ production. Key results show the water-to-carbon ratio and ESR temperature contributing 59.22% and 32.69% to production conditions’ impact, respectively. Graphical and mathematical models validate these findings. Moving forward, further experimental validation of optimal conditions for multi-response gas generation is recommended. This study pioneers a transformative approach towards sustainable, environmentally friendly H_2_ production.

## Introduction

Fossil fuel emissions clog our skies, staining the very air we breathe with pollution. The relentless plunder of non-renewable resources only deepens this ecological wound, driving urgent calls for a shift towards sustainable alternatives. Enter hydrogen is a promising contender in the battle against environmental degradation (Bolt et al. 2020; Karaoglu and Yolcular 2022). With its lofty energy density, pristine purity, and infinite regenerative potential, hydrogen emerges as a beacon of hope amidst the murky landscape of energy alternatives (Satyapal et al. 2007). Yet, harnessing its power demands a unique understanding—for hydrogen marches to the beat of its chemical drum (Karaoglu and Yolcular 2022). When unleashed within a fuel cell, hydrogen dances as a pristine performer, emitting naught but water vapor. Its celestial cleanliness positions it as the darling of power generation and transportation, offering portable electricity solutions for both vehicles and dwellings alike (Kumar and Muthukumar 2020; Xiao et al. 2022). The allure of clean, secure energy beckons from the hydrogen horizon, tantalizing us with the promise of a brighter tomorrow. With its prowess to fuel both fuel cells and internal combustion engines, hydrogen stands are poised to revolutionize our energy landscape. But despite its cosmic abundance, hydrogen remains a reluctant guest on our planet’s surface, necessitating innovative production and storage solutions to unlock its full potential. To weave hydrogen into the fabric of our sustainable future, we must harvest its power from renewable sources, embracing techniques that are gentle on both our planet and our wallets. Only then can we truly harness the boundless energy of hydrogen, ushering in an era where clean, green power reigns supreme.

In a city battling pollution, two solutions emerged: carbon capture and utilization (CCU) (Dou et al. 2023) and carbon reduction optimization (CRO). CCU captured emissions from factories, repurposing them for various uses. Meanwhile, CRO-optimized processes reduce emissions at their source. While CCU seemed effective, CRO’s proactive approach proved superior. By targeting emissions directly, CRO minimized the need for extensive infrastructure and promoted sustainable practices. Ultimately, CRO led to cleaner air and a brighter future for the city. In the expansive realm of sustainable energy, an array of diverse endeavors has been undertaken to grapple with the pressing issue of emissions during hydrogen production from traditional sources such as WGSR and ESR. Casanovas et al. (2009) conducted a comprehensive study delving into the investigation of Mn promotion over Co/ZnO catalysts for ethanol steam reforming (ESR) and water gas shift (WGS) reactions, unraveling intricate insights into catalyst characteristics and performance. Nevertheless, amidst its elucidation, a conspicuous lacuna emerges, as it overlooks the crucial aspect of optimizing hydrogen production and minimizing carbon emissions, thereby constituting a significant drawback. This oversight underscores missed opportunities to enhance process efficiency and bolster environmental sustainability. On a similar note, Sharma et al. (2017) eloquently underscored ethanol steam reforming’s potential for hydrogen production, yet astutely noted a glaring absence in optimization for hydrogen yield and reduction of carbon emissions, accentuating the exigency for more holistic strategies. Additionally, Chen et al. (2017) singularly focused on hydrogen generation from ESR and WGSR techniques, regrettably sidestepping the imperative of addressing carbon emission reduction, thus magnifying environmental apprehensions. This recurrent theme of neglecting carbon emission mitigation alongside hydrogen yield optimization, as exemplified in Quan et al.’s (2024) evaluation of Ni–Ce/mesopore Y catalysts for ESR, speaks volumes about the prevailing trend in the literature. Furthermore, Cordaro et al.’s (2024) proposition of a model for co-generating hydrogen and electricity unveils a pertinent concern—its oversight in optimizing both hydrogen and carbon emissions, underscoring an inherent deficiency in overall efficiency and sustainability considerations. While Di Nardo et al. (2024) provided a nuanced analysis of CO_2_ emissions across diverse steam reforming processes, their failure to optimize hydrogen yield and carbon emissions is a palpable shortcoming that merits attention. Similarly, Kim et al.’s (2024) exploration into enhancing hydrogen production from waste-derived syngas signals a clear imperative for further optimization in both hydrogen yield and carbon minimization. Ovalle-Encinia and Lin’s (2024) investigation of a CO_2_-perm-selective membrane reactor for WGS reaction, albeit demonstrating enhanced CO conversion and H_2_ purity, notably disregarded optimization for hydrogen yield and carbon emissions, thus presenting an area ripe for improvement in reactor efficiency and sustainability. Against this backdrop, our study endeavors to bridge this gap by integrating comprehensive strategies aimed at minimizing emissions and maximizing hydrogen yield through both singular (Taguchi design) and multi-response (grey) optimization analyses, thereby contributing substantively to a more environmentally benign and economically sustainable paradigm in hydrogen production.

The Taguchi design methodology has proven effective in reducing experiment variability and improving the quality and efficiency of processes, products, and systems (Abifarin 2021; Roy 2010). It is commonly utilized to optimize process or production parameters for enhanced performance characteristics (Taguchi 1995). However, a limitation of Taguchi design is its inability to optimize multiple performance characteristics simultaneously (Abifarin et al. 2021a). When dealing with multiple characteristics, complications arise as they may not align in the same domain and may have conflicting objectives. To address the challenges of optimizing multiple performance characteristics, grey relational analysis (GRA) has been extensively integrated with the Taguchi design technique across various applications, including automotive (Abifarin and Ofodu 2022a; Garud and Lee 2023; Salmani et al. 2022), aerospace (Panwar and Chandna 2023; Unnikrishna Pillai et al. 2023), biomedical (Abifarin et al. 2023a,b, 2022; Hussain et al. 2023), CNC machining (Aravind et al. 2017; Esangbedo and Abifarin 2022), materials fabrication (Esangbedo and Abifarin 2023; Abifarin 2021; Tzeng et al. 2009), and energy production (Deepanraj et al. 2017; Kadier et al. 2015; Abifarin and Ofodu 2022b; Shi et al. 2023; Vasantharaj et al. 2017). The optimization results obtained through this approach are effective and efficient. Despite the exceptional advantages of Taguchi GRA in optimizing multiple performance characteristics, no study, to the best of our knowledge, has focused on maximizing hydrogen production while minimizing carbon emissions from carbon-sourced hydrogen. This paper presents a model and settings for hydrogen production from any carbon-based hydrogen source, aiming to reduce carbon emissions and increase hydrogen energy for environmental sustainability and human consumption.

In conclusion, while the literature has extensively explored methods such as water–gas shift (WGS) reaction and ethanol steam reforming for hydrogen production, a notable gap remains in the simultaneous optimization of hydrogen yield and carbon emissions reduction. Our work stands out by addressing this gap and introducing novel strategies to integrate Taguchi design and grey relational analysis. By leveraging these techniques, we aim to maximize hydrogen production while minimizing carbon emissions from carbon-sourced hydrogen. This approach represents a significant advancement in the field, offering a more comprehensive and sustainable solution to the challenges of hydrogen production. Through our study, we not only bridge the gap in the existing literature but also contribute to a cleaner, greener energy landscape, paving the way for a more environmentally friendly and economically viable approach to hydrogen production.

## Methodology

### Taguchi design analysis

The data analyzed in this study is derived from the study of Chen et al. (2017), and here is the summary of the conditions employed to obtain the data from their work: The system employed included the experimentation of producing hydrogen and enriching carbon dioxide. It included a feeding unit, a reaction unit, a condenser, and units for gas and liquid analysis. Ethanol–water mix in a flask was fed using a pump. The reaction unit had ESR and WGSR reactors, with controlled temperatures. ESR used a Ni-based catalyst, with liquid hourly space velocity (LHSV) values of 2.14 and 4.28 h^−1^. WGSR used a Fe–Cr-based catalyst. Gas from ESR went into WGSR. Gas and liquid analyses were done using specific instruments. Calibration ensured precise measurements. Each experiment lasted 4 h, yielding steady results.

**Fig. 1:**
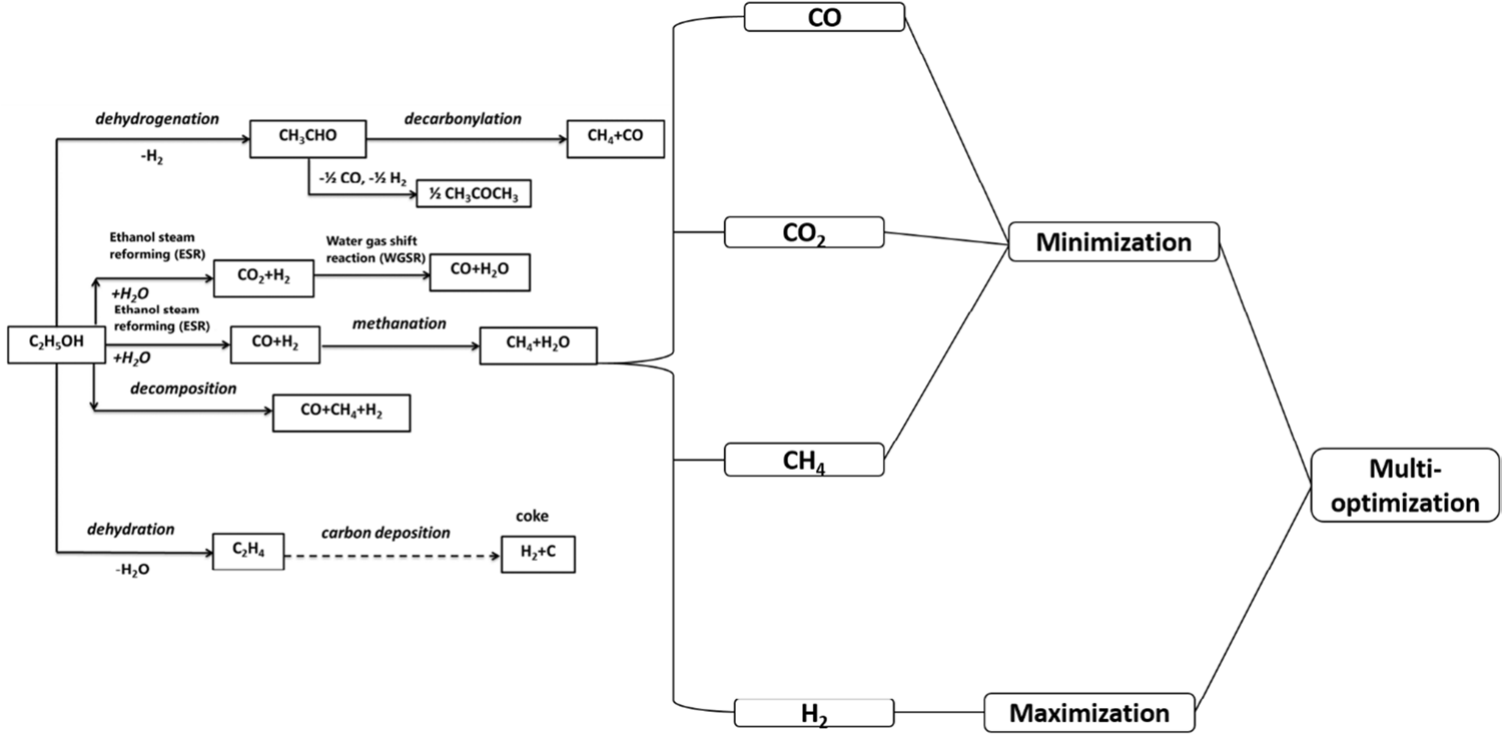
Singular and multi-response gases design analytical procedure

For the trials, a Taguchi L9 orthogonal array (OA) was used, comprising nine runs as indicated in Table 1. Four response factors, CO, CO_2_, CH_4_, and H_2_, were used to evaluate the gases produced, as shown in Table 1. The results and models generated for the four gases are presented and discussed in “Results and discussion.” Like the study of Abifarin and Owolabi (2023), note that the production conditions employed are labeled A to D (Table 1) for the mathematical modeling of resultant gases discussed in “Results and discussion.” The design overview is depicted in Fig. 1.

**Table 1:** Orthogonal array design

Design run	Hydrogen production conditions from ethanol steam reforming and water gas shift reaction pathways				Generated gases as responses			
	A: Rate of feed liquid flow (mL/min)	B: Water-to-carbon ratio	C: ESR temperature (°C)	D: WGSR temperature (°C)	CO (%)	CO_2_ (%)	CH_4_ (%)	H_2_ (%)
1	2	3	400	300	1.62	21.82	6.04	66.71
2	2	4	450	350	0.48	21.18	6.07	66.81
3	2	5	500	400	0.67	23.44	1.58	70.38
4	3	3	450	400	1.85	21.96	3.6	68.69
5	3	4	500	300	1.44	21.3	1.52	70.43
6	3	5	400	350	1.14	21.25	2.75	69.51
7	4	3	500	350	3.69	18.99	4.31	67.67
8	4	4	400	400	1.23	21.52	3.29	67.53
9	4	5	450	300	2.8	20.51	1.62	70.73

### Multi-gas response optimization using GRA

The Taguchi design of experiments (DOE) method is adequate for evaluating optimal processing parameters for a single performance response. However, when optimizing two or more responses, a multi-objective optimization technique is necessary. Grey relational analysis (GRA) is employed to handle irregular finite data by normalizing all four gases into a single domain for Taguchi optimization analysis. Therefore, multi-gas optimization of processing parameters is conducted using the Taguchi GRA technique.

#### Grey relational analysis

The preprocessing of the four experimental gas data was done to ensure that they fell between zero and one, as stated in the grey relational analysis technique. The “higher-the-better” (Eq. 1) preprocessing data methodology was applied to H_2_, whereas the “smaller-the-better” method was applied to CO, CO_2_, and CH_4_ (Eq. 2). The reason for this choice is that a higher quantity of H_2_ is desired for fuel efficiency maximization, while as small as possible CO, CO_2_, and CH_4_ emissions are desired for emission reduction (Len and Luque 2023; Zurrer et al. 2023). Table 3 shows the preprocessed data (x*_i_), which is commonly referred to as grey relational generation. For each of the four generated gases, an ideal sequence, *x*_0_ (*k*) (*k* = 1, 2,… 9), was compared. The grey relational coefficient was then processed by computing the deviation sequence (Eq. 3). Table 3 also displays the deviation sequence (∆_*oi*_) for each of the four experimental gases. Next, using Eq. 4, the grey relational coefficient (GRC) was calculated. The link between the expected and actual experimental data is depicted by the grey relational coefficient. Additionally, it provides the ability to link and calculate the statistical means of the different experimental data that are not additive. The five grey relational coefficients were then averaged to determine the grey relational grade or GRG, in Eq. 5. Table 2 also displays the GRG and GRC values. The total reaction of the four experimental gases is provided by the grey relational grade. Stated differently, the unique answer (GRG) produced by the grey computational analysis makes the hitherto impractical optimization of the four complex data possible. The level exhibiting the highest GRG is the optimization of the material’s design parameters.

**Table 2:** Grey relational analytical results

Design run	Grey relational generation (x*_i_)				Deviation sequence ($\Delta$_*oi*_)				Grey relational coefficient (${\varepsilon }_{i}$ (*k*))				GRG (${\gamma }_{i}$)
	CO	CO_2_	CH_4_	H_2_	CO	CO_2_	CH_4_	H_2_	CO	CO_2_	CH_4_	H_2_	
1	0.645	0.364	0.007	0	0.355	0.636	0.993	1	0.585	0.440	0.335	0.333	0.423
2	1	0.508	0	0.025	0	0.492	1	0.975	1	0.504	0.333	0.339	0.544
3	0.941	0	0.987	0.913	0.059	1	0.013	0.087	0.894	0.333	0.974	0.852	0.763
4	0.573	0.333	0.543	0.493	0.427	0.667	0.457	0.508	0.54	0.428	0.522	0.496	0.497
5	0.701	0.481	1	0.925	0.299	0.519	0	0.075	0.626	0.491	1	0.870	0.747
6	0.794	0.492	0.730	0.697	0.206	0.508	0.270	0.304	0.709	0.496	0.649	0.622	0.619
7	0	1	0.387	0.239	1	0	0.613	0.761	0.333	1	0.449	0.397	0.545
8	0.766	0.431	0.611	0.204	0.234	0.569	0.389	0.796	0.682	0.468	0.562	0.386	0.524
9	0.277	0.658	0.978	1	0.723	0.342	0.022	0	0.409	0.594	0.958	1	0.740

1 $${x}_{i}\left(k\right)=\frac{{y}_{i}\left(k\right)-\text{min}{y}_{i}(k)}{{\text{max} y}_{i}\left(k\right)-\text{min}{y}_{i}(k)}$$

2 $${x}_{i}\left(k\right)=\frac{\text{max}{y}_{i}\left(k\right)-{y}_{i}\left(k\right)}{{\text{max} y}_{i}\left(k\right)-\text{min}{y}_{i}(k)}$$

3 $${\Delta }_{oi}\left(k\right)=\Vert {x}_{0}\left(k\right)-{x}_{i}(k)\Vert$$

4 $${\xi }_{i}\left(k\right)=\frac{{\Delta }_{\text{min}}+\zeta {\Delta }_{\text{max}}}{{\Delta }_{oi}\left(k\right)+\zeta {\Delta }_{\text{max}}}$$

5 $${\gamma }_{i}=\frac{1}{n}\sum_{i=1}^{n}{\xi }_{i}\left(k\right)$$

In the equation, where *y*_*i*_ (*k*) represents the starting sequence of the response mean and *x*_*i*_ (*k*) stands for the preprocessed data for the ith experiment. The sequences of deviation, reference, and comparability are denoted by Δ_*oi*_ (*k*), *x*_*o*_ (*k*), and *x*_*i*_ (*k*), respectively. The GRC value of each gas response, calculated as a function of the minimum and maximum deviations of each response variable, Δ_min_ and Δ_max_, is reflected in ξ_*i*_ (*k*). The differentiating coefficient (0∼1) is ζ; however, each parameter is often given a separate weight of 0.5. The value of GRG for the *i*th experiment is represented by *γ*_*i*_, where *n* is the total number of performance characteristics.

### Variance analysis

The purpose of ANOVA is to determine whether the processing factors in the experiment design (DOE) have a substantial impact on the production efficiency being evaluated. The analysis of interactions between processing factors and their impact on dependent variables has also extensively utilized the ANOVA table (Sambasevam et al. 2023; Abifarin et al. (2021b)). It is employed to investigate the amount of contribution of production factors to the efficiency of the whole system. Here, we used ANOVA with Minitab 16 software to investigate the significance and contribution of production factors (rate of feed liquid flow, H_2_O/C ratio, ESR temperature, WGSR temperature) on the individual response gases and their multi-gas characteristics.

## Results and discussion

### Hydrogen (H_2_) maximization analysis

#### Significance of hydrogen production conditions on hydrogen generation

The results from Table 3 highlight the significant influence of various hydrogen production conditions on the yield of H_2_. Specifically, the water-to-carbon ratio emerges as the most influential factor, contributing 50.88% to the maximization of H_2_ yield. This underscores the importance of maintaining an optimal ratio of water to carbon for efficient hydrogen production in ethanol steam reforming (ESR) and water gas shift reaction (WGSR) pathways. Additionally, the rate of feed liquid flow and ESR temperature each contribute approximately 18% to H_2_ yield, indicating their substantial impact on the process. Conversely, WGSR exhibits the lowest contribution, accounting for only 12.69% of H_2_ yield variation. These findings emphasize the necessity of prioritizing the water-to-carbon ratio as a key production condition for maximizing hydrogen output in ESR and WGSR processes. The effectiveness of the model is further confirmed by a residual error of zero, validating its accuracy in predicting H_2_ generation under varying production conditions. Equation 6 mathematically represents the relationship between production conditions and H_2_ yield, with variables *A*, *B*, *C*, and *D* denoting the resultant H_2_ values corresponding to feed liquid flow rate, water-to-carbon ratio, ESR temperature, and WGSR temperature, respectively. This comprehensive analysis elucidates the critical role of process parameters in influencing hydrogen production efficiency and underscores the importance of optimizing conditions to enhance overall process performance.

**Table 3:** Analysis of variance of hydrogen production conditions on the generation of hydrogen

Factor	Degree of freedom	Adjusted sum square	Adjusted mean square	Contribution (%)
Rate of feed liquid flow (mL/min)	2	3.7538	1.87688	18.27
Water-to-carbon ratio	2	10.4572	5.22861	50.88
ESR temperature (°C)	2	3.7318	1.86588	18.16
WGSR temperature (°C)	2	2.6088	1.30441	12.69
Residual error	0	0	0	-
Total	8	-	10.27578	100

6 $${\text{H}}_{2} \left(\%\right)=55.69+0.338A+1.25B+0.0158C-0.00029D$$

#### Effect of hydrogen production conditions on hydrogen production

**Fig. 2:**
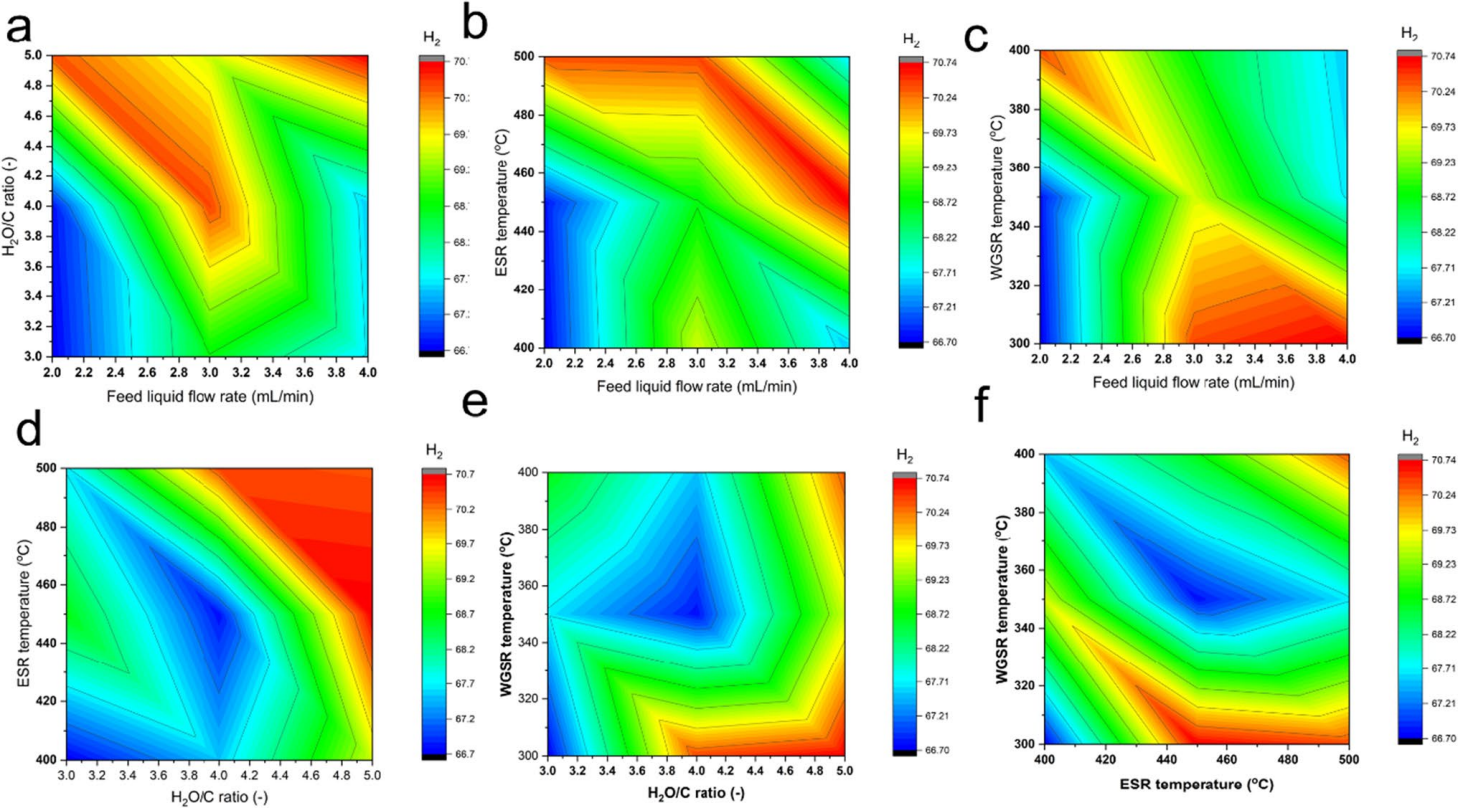
Interaction between the production parameters on hydrogen (H_2_) production

The interaction between hydrogen production conditions and their impact on H_2_ generation is crucial for maximizing production efficiency. Illustrated in Fig. 2, the varying shades from deep red to deep blue denote the spectrum of H_2_ levels, with deep red representing the highest and deep blue the lowest. Ideally, production conditions yielding a deep red hue indicate optimal H_2_ generation. Consequently, a higher water-to-carbon (H_2_O/C) ratio and ESR temperature, in conjunction with other hydrogen production conditions, lead to maximal H_2_ production. Conversely, lower values of feed liquid flow rate and WGSR temperature contribute to optimal H_2_ yield, while also offering energy-saving benefits. Therefore, by precisely controlling production conditions as guided by the model depicted in Fig. 2 and Eq. 6, maximum hydrogen production can be achieved via ethanol steam reforming and water gas shift reaction pathways. This underscores the importance of strategic process parameter management in enhancing H_2_ generation efficiency and overall process performance.

### Carbon monoxide (CO) reduction analysis

#### Significance of hydrogen production conditions on carbon monoxide

After analyzing the graphical representation depicting the interaction of various hydrogen production conditions on CO gas generation, it is imperative to quantitatively assess the effects of these production conditions on CO generation during hydrogen production from ethanol steam reforming and water gas shift reaction pathways. Utilizing ANOVA analysis, Table 4 provides insights into the percentage contribution of each production condition to CO generation. The results highlight the significant impact of the rate of feed liquid flow, contributing 50.77%, followed by the water-to-carbon ratio at 32.95%. Remarkably, the residual error exhibits zero contribution, indicating the model’s exceptional goodness of fit. Regression modeling facilitated the development of a mathematical model for CO generation, as presented in Eq. 7, where variables *A*, *B*, *C*, and *D* correspond to the resultant generated values of CO gas concerning the rate of feed liquid flow, H_2_O/C ratio, ESR temperature, and WGSR temperature, respectively. This comprehensive analysis underscores the critical role of production conditions in influencing CO generation, providing valuable insights for optimizing hydrogen production pathways while minimizing unwanted byproducts like CO.

**Table 4:** Analysis of variance of hydrogen production conditions on carbon monoxide

Hydrogen production conditions	Degree of freedom	Adjusted sum square	Adjusted mean square	Contribution (%)
Rate of feed liquid flow (mL/min)	2	4.23136	2.11568	50.77
Water to carbon ratio	2	2.74602	1.37301	32.95
ESR temperature (°C)	2	0.55829	0.27914	6.70
WGSR temperature (°C)	2	0.79869	0.39934	9.58
Residual error	0	0	0	0
Total	8	8.33436	4.16717	100

7 $$\text{CO }\left(\%\right)=0.63+0.825A-0.425B+0.00603C-0.00703D$$

#### Effect of hydrogen production conditions on carbon monoxide reduction

**Fig. 3:**
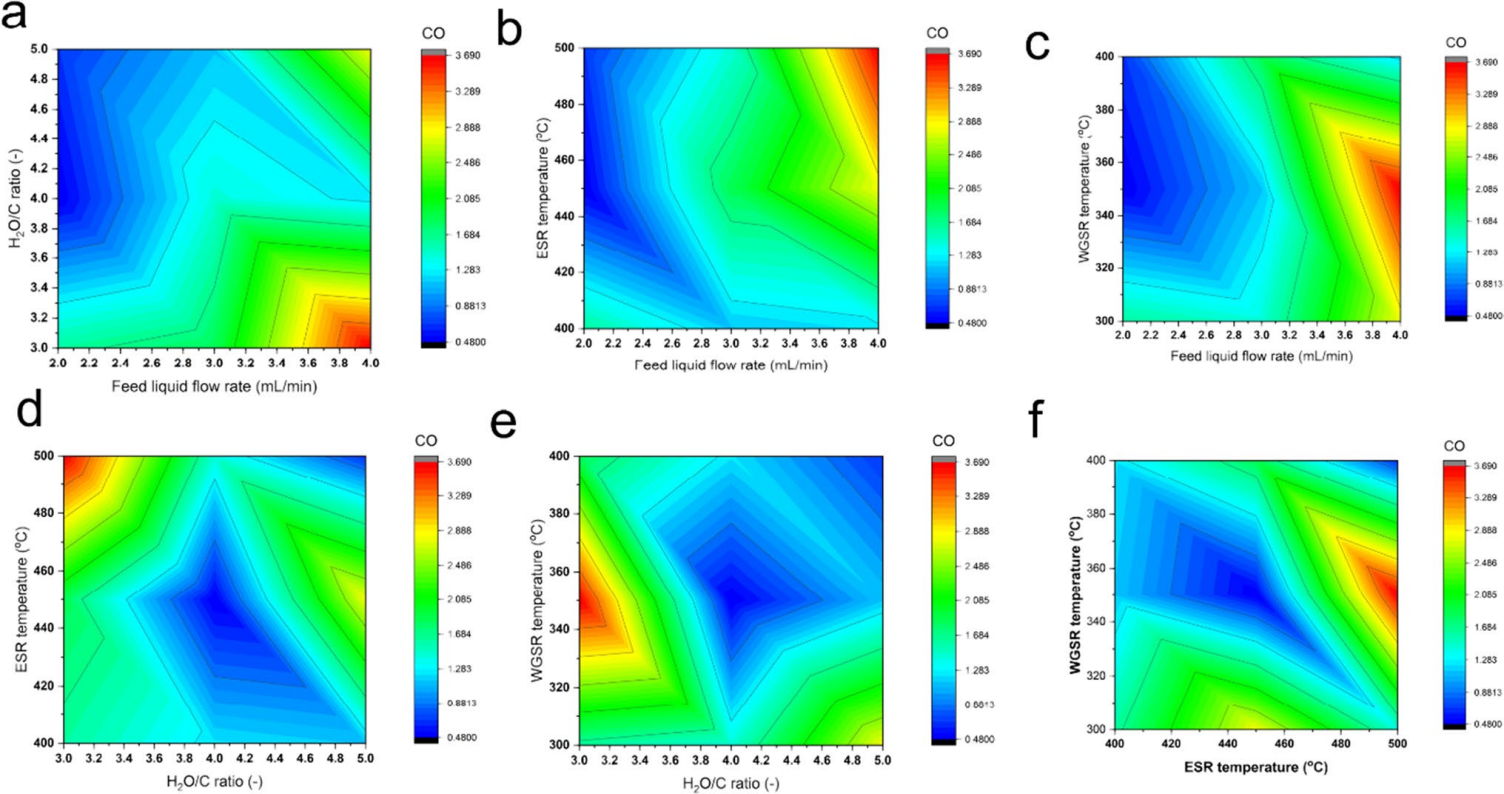
Interaction between the production parameters on carbon monoxide reduction

To explore the impact of hydrogen production conditions on CO generation, Origin Pro software was employed to simulate the interaction among various production parameters. Figure 3 presents the emitted percentages of CO relative to the interactions of production conditions, with different color gradients indicating varying CO levels. Notably, deep red hues signify higher CO levels, while deep blue hues denote lower CO levels. Overall, the results reveal that a high water-to-carbon (H_2_O/C) ratio coupled with a lower rate of feed liquid flow tends to result in lower CO generation when interacting with other production conditions. Interestingly, the figure illustrates that different ESR and WGSR temperatures can minimize CO generation. Specifically, employing lower temperatures, such as 400 °C for ESR and 300 °C for WGSR, can yield minimal CO generation, suggesting relatively low energy costs required to mitigate CO production. This graphical modeling for CO generation offers valuable insights for researchers and production engineers, enabling them to manipulate various production conditions to minimize CO generation when producing hydrogen via ethanol steam reforming and water gas shift reaction pathways.

### Carbon dioxide (CO_2_) reduction analysis

#### Significance of hydrogen production conditions on the generation of carbon dioxide

Like the CO analysis, ANOVA was utilized to explore the effect and significance of the employed hydrogen production conditions on CO_2_ generation, as depicted in Table 5. It is evident that WGSR temperature significantly influences CO_2_ generation during hydrogen production, with a contribution of 45.18%, slightly higher than the contribution (44.51%) of the rate of feed liquid flow, which contrasts with the case of CO generation. Furthermore, the results indicate that the water-to-carbon ratio has a lesser effect (8.71%) compared to CO generation. The zero residual error also reflects the goodness of fit of the model. Additionally, like CO generation, varying production conditions yield different CO_2_ values, as mathematically presented in Eq. 8, where *A*, *B*, *C*, and *D* represent the resultant generated values of CO_2_ gas for the rate of feed liquid flow, H_2_O/C ratio, ESR temperature, and WGSR temperature, respectively.

**Table 5:** Analysis of variance of hydrogen production conditions on carbon dioxide

Factor	Degree of freedom	Adjusted sum square	Adjusted mean square	Contribution (%)
Rate of feed liquid flow (mL/min)	2	5.03127	2.51563	44.51
Water to carbon ratio	2	0.9842	0.4921	8.71
ESR temperature (°C)	2	0.18107	0.09053	1.6
WGSR temperature (°C)	2	5.10647	2.55323	45.18
Residual error	0	-	-	-
Total	8	-	5.65149	100

8 $${\text{CO}}_{2} \left(\%\right)=19.87-0.903A+0.405B+0.00287C+0.01097D$$

#### Effect of hydrogen production conditions on the generation of carbon dioxide

**Fig. 4:**
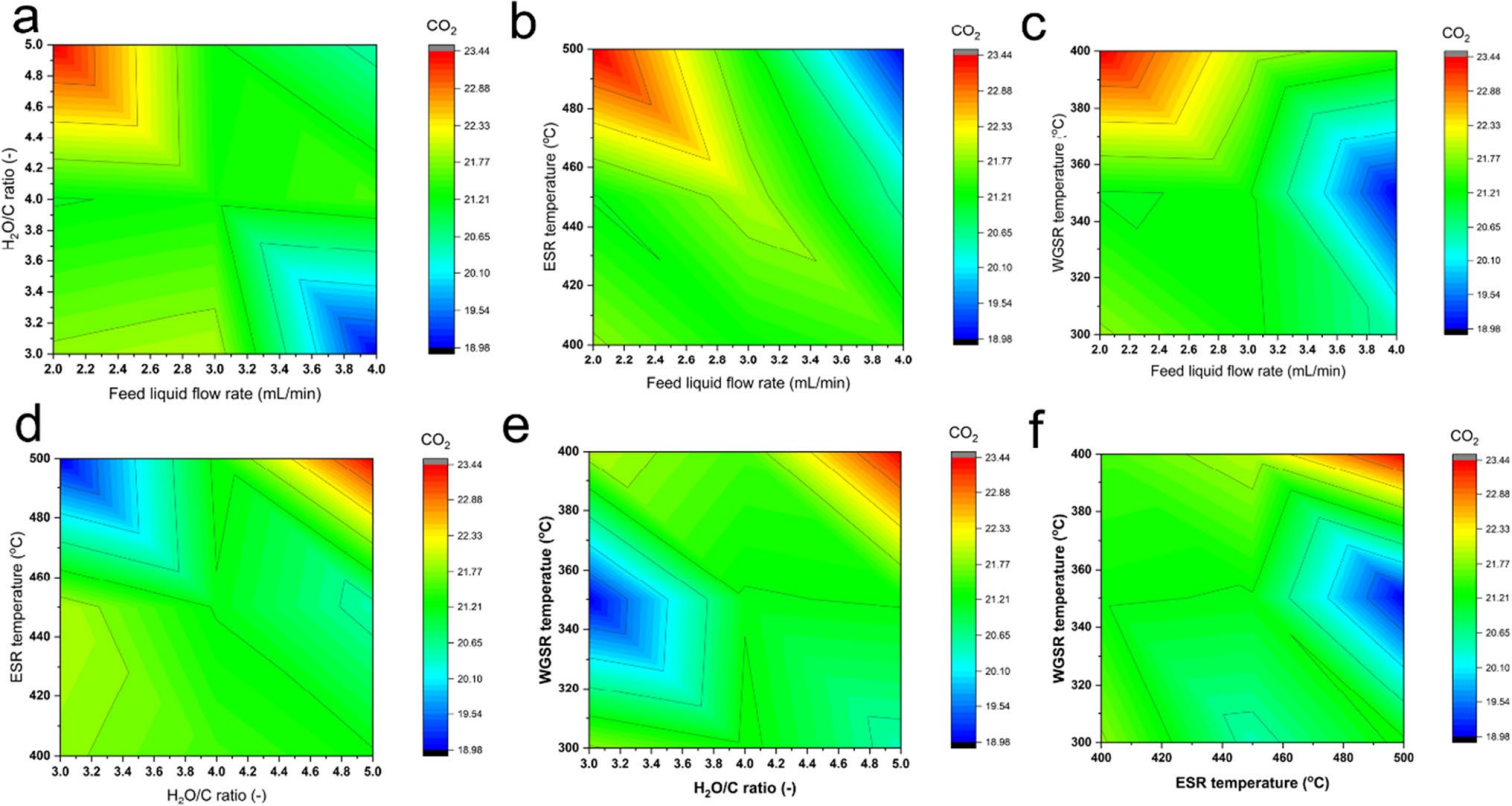
Interaction between the production parameters on carbon dioxide reduction

Similar to the CO reduction analysis, the effect of hydrogen production conditions on CO_2_ generation was investigated, and the interaction between these conditions on the resulting percentage of CO_2_ was simulated, as shown in Fig. 4. The different color outlets reflect variations in CO_2_ percentages, with deep red indicating the highest level of CO_2_ and deep blue indicating the lowest level. In contrast to CO generation, when a low value of water-to-carbon (H_2_O/C) ratio and a high value of the rate of feed liquid flow interact with other production conditions, lower CO_2_ generation is observed. Additionally, a higher value of ESR temperature results in lower CO_2_ generation. The results further reveal that WGSR temperature in the range of 320 to 370 °C yields relatively low CO_2_ levels, indicating a lesser WGSR energy cost to produce hydrogen with minimal CO_2_ generation. These modeled production conditions presented in the figure provide a pathway to optimizing lower CO_2_ generation during hydrogen production from ethanol steam reforming and water gas shift reaction pathways.

### Methane (CH_2_) minimization analysis

#### Significance of hydrogen production conditions on the generation of methane

The effect and significance of the employed hydrogen production conditions on generated CH_4_ were analyzed, as presented in Table 6. The results indicate that the water-to-carbon ratio is the most significant production condition for minimizing CH_4_, contributing 42.73%, followed by the rate of feed liquid flow, with a contribution of 24.34%. Interestingly, both ESR and WGSR show approximately the same contribution (16%). This analysis underscores the importance of all four hydrogen production conditions in minimizing CH_4_ during the production of hydrogen from the employed pathways. The model’s reliability is confirmed by a residual error of zero percentage. Furthermore, different CH_4_ values can be obtained when varying the production conditions, as mathematically presented in Eq. 9, where *A*, *B*, *C*, and *D* represent the resultant generated values of CH_4_ gas for the rate of feed liquid flow, H_2_O/C ratio, ESR temperature, and WGSR temperature, respectively.

**Table 6:** Analysis of variance of hydrogen production conditions on methane

Factor	Degree of freedom	Adjusted sum square	Adjusted mean square	Contribution (%)
Rate of feed liquid flow (mL/min)	2	6.1862	3.0931	24.34
Water to carbon ratio	2	10.8589	5.42943	42.73
ESR temperature (°C)	2	4.1653	2.08263	16.39
WGSR temperature (°C)	2	4.2025	2.10123	16.54
Residual error	0	0	0	0
Total	8	-	12.70639	100

9 $${\text{CH}}_{2} \left(\%\right)=18.82-0.745A-1.333B-0.0156C-0.0024D$$

#### Effect of hydrogen production conditions on the generation of methane

**Fig. 5:**
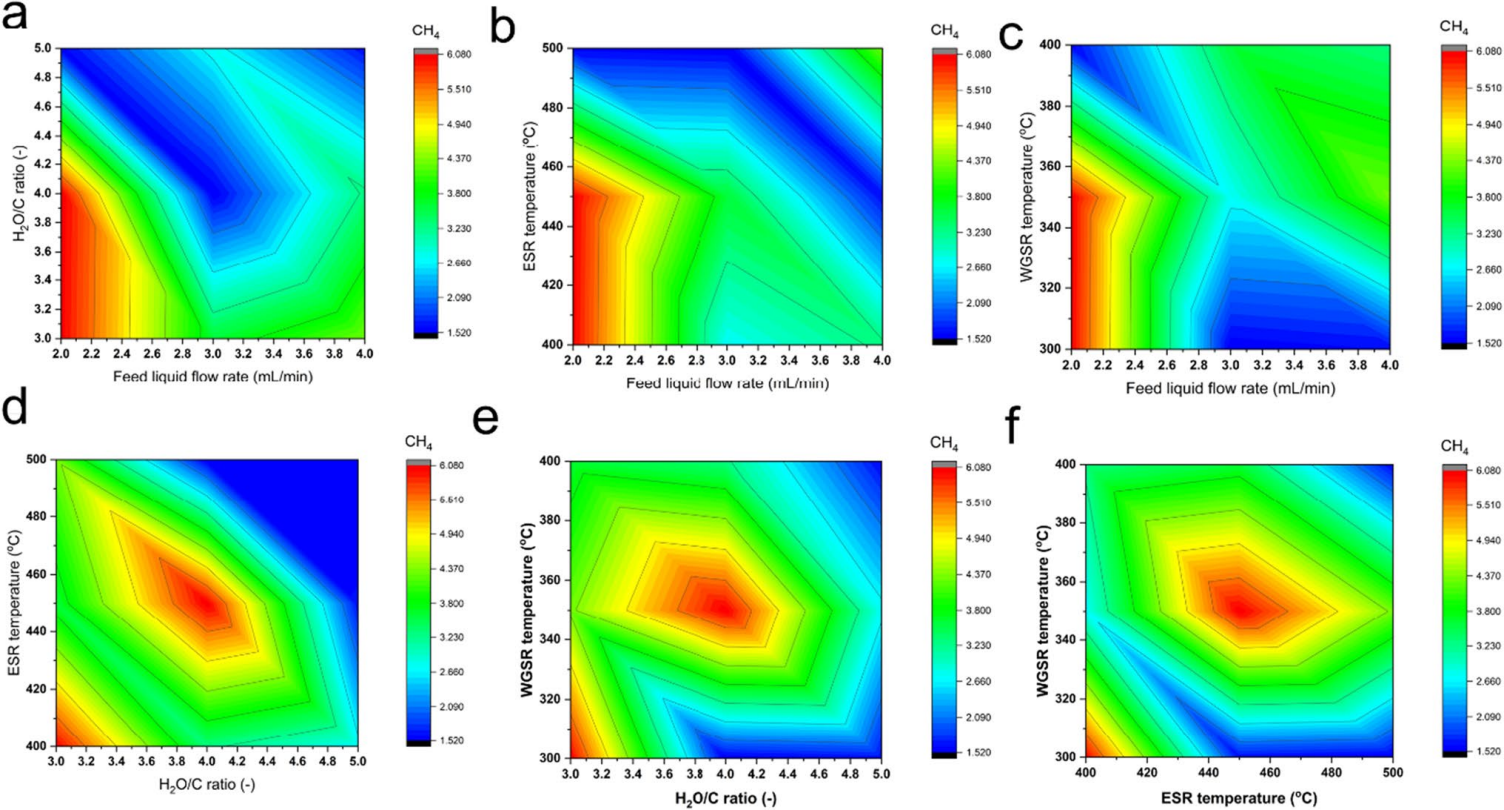
Interaction between the production parameters on methane (CH_4_) production

A similar analysis was conducted to assess the effect of hydrogen production conditions on the generation of CH_4_, with the interaction between these conditions simulated and presented in Fig. 5. Different color outlets in the figure represent variations in CH_4_ percentages, where deep red indicates the highest level of CH_4_ and deep blue indicates the lowest. It was observed that higher values of the water-to-carbon (H_2_O/C) ratio and ESR temperature when interacting with other hydrogen production conditions result in the minimization of CH4. Additionally, different values of the rate of feed liquid flow interacting with other production conditions can also lead to the minimization of CH_4_. This suggests that a lower liquid flow rate (for energy cost minimization) can be applied to minimize CH_4_ generation during the production of hydrogen from ethanol steam reforming and water gas shift reaction pathways. Interestingly, it was found that both lower and higher WGSR temperature ranges can yield minimum CH_4_ values. However, to save on hydrogen production costs and minimize CH_4_, a WGSR temperature as low as 300 °C is recommended. This graphical model aids in decision-making regarding CH_4_ minimization during hydrogen production from ethanol steam reforming and water gas shift reaction pathways.

#### Singular response optimization analysis on hydrogen production conditions on the resultant gases

**Fig. 6:**
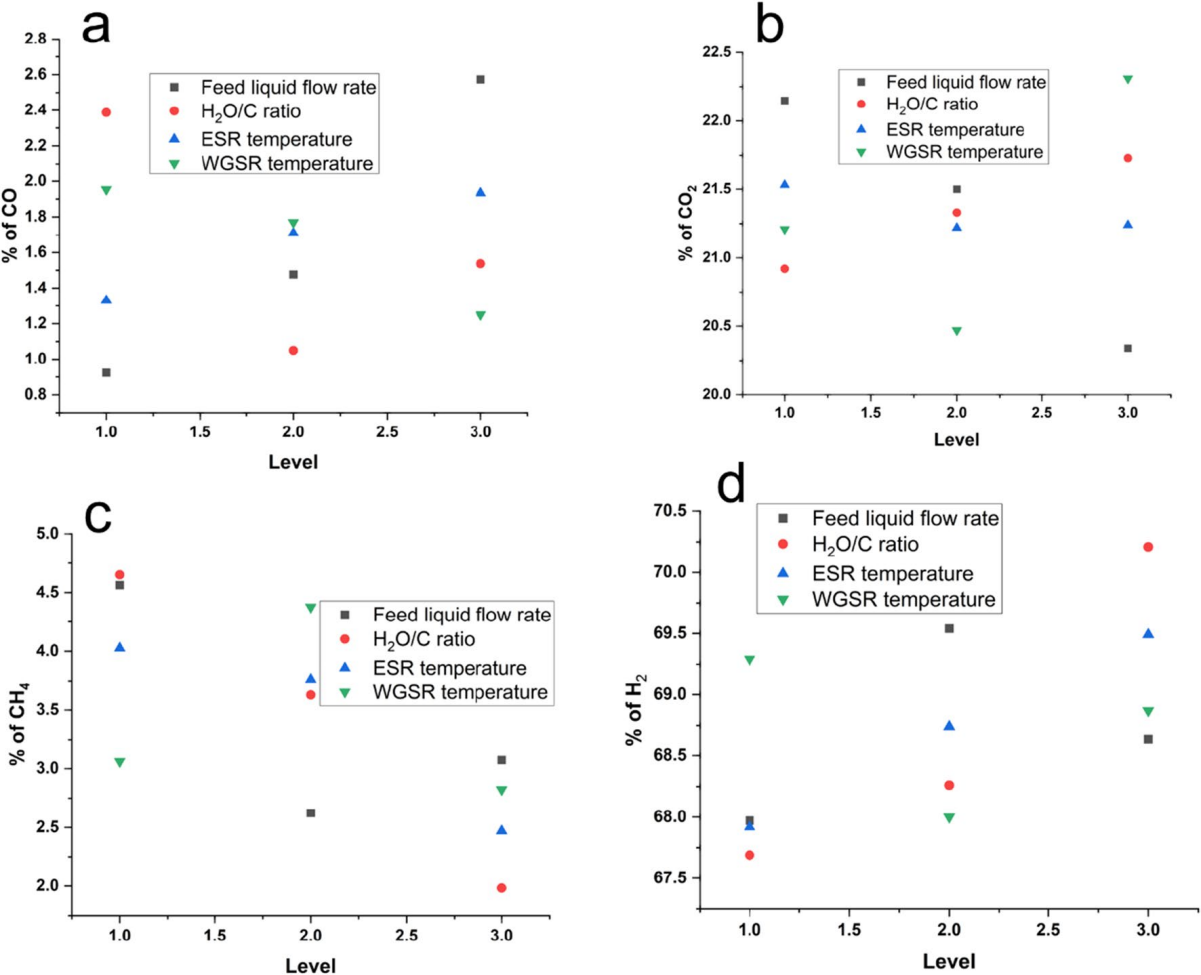
The effect of hydrogen production conditions on individual performance responses. **a** Carbon monoxide (CO). **b** Carbon dioxide (CO_2_). **c** Methane (CH_4_). **d** Hydrogen (H_2_)

The resultant gases outlined in Table 1 underwent Taguchi design analysis for individual response optimization, as depicted in Fig. 6. This analysis distinctly illustrates the production conditions yielding optimal gases, aiming to minimize undesired gases (CO, CO_2_, and CH_4_) while maximizing the desired gas (H_2_). Figure 6a showcases the optimal conditions for minimizing CO gas, revealing a rate of feed liquid flow of 2 mL/min, water-to-carbon ratio of 4, ESR temperature of 400 °C, and WGSR temperature of 300 °C. Figure 6b illustrates CO_2_ minimization, indicating optimal conditions of a liquid flow rate of 4 mL/min, water-to-carbon ratio of 3, ESR temperature of 500 °C, and WGSR temperature of 350 °C. For methane reduction, Fig. 6c displays optimal conditions with a liquid flow rate of 3 mL/min, water-to-carbon ratio of 5, ESR temperature of 500 °C, and WGSR temperature of 400 °C. Lastly, Fig. 6d reveals the maximization of hydrogen production at optimal conditions of a liquid flow rate of 2 mL/min, water-to-carbon ratio of 3, ESR temperature of 400 °C, and WGSR temperature of 350 °C. These observations highlight different optimal production conditions for the resultant gases, complicating conclusions on singular responses given the undesirability of carbon-based gases and the desirability of hydrogen. Consequently, the subsequent section delves into the results presented with Taguchi grey relational analysis, which effectively addresses this complexity and indecision challenge.

#### Multi-response optimization analysis on hydrogen production conditions on the resultant gases

**Fig. 7:**
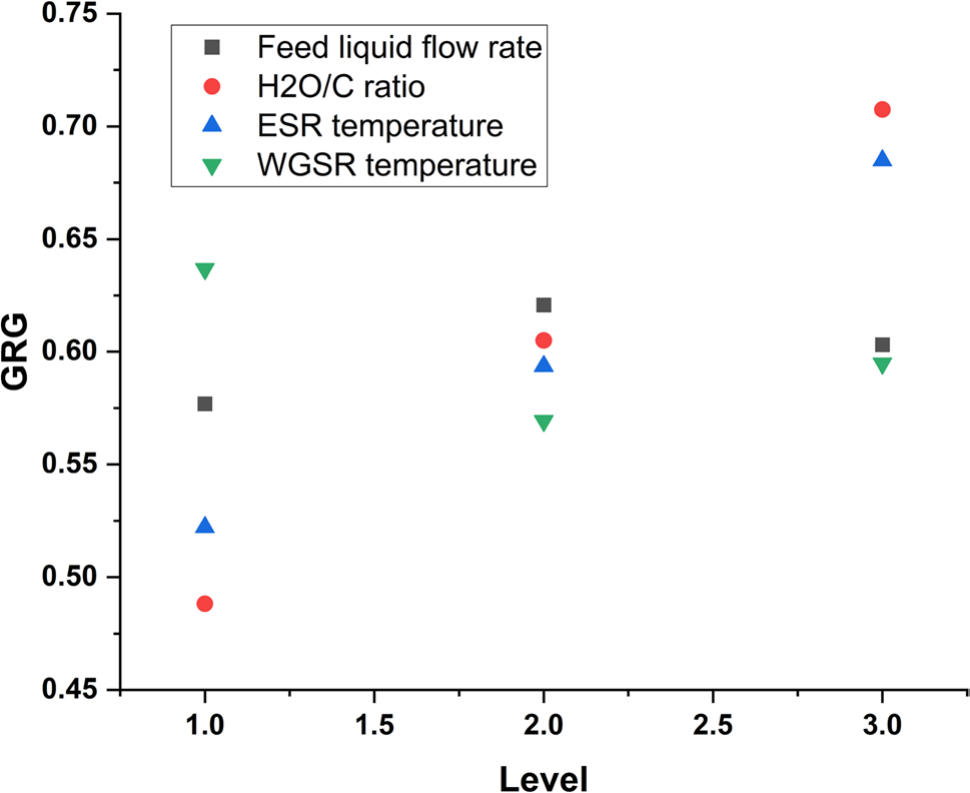
Effect of hydrogen production conditions on the multi-response gases (GRG)

Grey relational analysis (GRA) is commonly utilized to address problems characterized by limited information arrangements, particularly in scenarios with ambiguous frameworks lacking starkly contrasted layouts. Within the grey system, all data is represented by white, while the absence of data is depicted as black. Figure 7 presents a graphical representation of multi-response gases, aiming to minimize Co, CO_2_, and CH_4_ gases while maximizing hydrogen production simultaneously. The results indicate that the optimal hydrogen production conditions are a liquid flow rate of 2 mL/min, water-to-carbon ratio of 3, ESR temperature of 300 °C, and WGSR temperature of 350 °C. Intriguingly, these optimal conditions for multi-response gas optimization differ from those for individual optimization. Furthermore, they do not align with the design run employed in the work of Chen et al. (Chen et al. 2017). This discrepancy underscores the importance of conducting both singular and multi-response optimization analyses, serving as the primary motivation for this study.

To comprehensively assess the significance and percentage contribution of each production condition to the multi-response gases, an analysis of variance (ANOVA) was conducted for the grey relational grade (GRG) values at a confidence level of 95%, detailed in Table 7. The findings revealed that the water-to-carbon (H_2_O/C) ratio emerged as the most influential production condition, with a substantial contribution of 59.22%, followed closely by the ESR temperature at 32.69%. Notably, while the WGSR temperature and rate of feed liquid flow also demonstrated significance, their contributions were comparatively lower than those of the conditions. These results offer conclusive optimized hydrogen production conditions to minimize undesired gases and maximize desired gas (H_2_) production. These conditions are particularly recommended for hydrogen production via ethanol steam reforming and water gas shift reaction pathways. The observed absence of residual error in the GRA ANOVA underscores the high level of confidence in the developed model. It is worth noting that varying the output GRG values of individual production conditions can yield different proportions of GRG, as expressed in Eq. 10. *A*, *B*, *C*, and *D* represent the resultant generated values of GRG for the rate of feed liquid flow, H_2_O/C ratio, ESR temperature, and WGSR temperature, respectively.

**Table 7:** Analysis of variance of hydrogen production conditions on grey relational grade (GRG)

Factor	Degree of freedom	Adjusted sum square	Adjusted mean square	Contribution (%)
Rate of feed liquid flow (mL/min)	2	0.002922	0.001461	2.39
Water to carbon ratio	2	0.072267	0.036134	59.22
ESR temperature (°C)	2	0.039895	0.019947	32.69
WGSR temperature (°C)	2	0.006954	0.003477	5.70
Residual error	0	0	0	0
Total	8	0.122039	0.061019	100

10 $$\text{GRG}=-0.463+0.0131A+0.1097B+0.001627C-0.000419D$$

## Conclusion

In a groundbreaking endeavor, this study pioneers the application of multi-response Taguchi grey relational analysis to tackle the intricate challenges surrounding optimal hydrogen production conditions. With a dual aim of maximizing hydrogen yield while minimizing CO, CO_2_, and CH_4_ emissions for enhanced energy efficiency, sustainability, and cleanliness, this research delves deep into the interplay of various production parameters. Leveraging Origin Pro software, we meticulously model the interactions between these parameters and their impact on individual gas outputs as well as overall multi-response gas generation. Through rigorous regression modeling, we unveil mathematical models for each gas, offering invaluable insights into the intricate dynamics at play. By elucidating the diverse hydrogen production conditions conducive to optimized gas yields, our study empowers the application of grey relational analysis to derive conclusive conditions for minimizing undesirable emissions and maximizing H_2_ production via ethanol steam reforming and water gas shift reaction pathways. Notably, our findings pinpoint optimal production conditions—rate of feed liquid flow at 2 mL/min, water-to-carbon ratio at 3, ESR temperature at 300 °C, and WGSR temperature at 350 °C—as key drivers of clean and efficient hydrogen production. Highlighting the pivotal roles of water-to-carbon ratio and ESR temperature, which contribute 59.22% and 32.69% respectively, our research provides a roadmap for sustainable and high-yield hydrogen production. With our developed graphical and mathematical models demonstrating exceptional goodness of fit, underscored by zero residual error, this study heralds a new era of design reliability. Moving forward, we advocate for experimental validation of the obtained multi-response optimal conditions, paving the way for transformative advancements in hydrogen production technology.
